# Neuroprotective effects of curcumin on the cerebellum in a rotenone‐induced Parkinson’s Disease Model

**DOI:** 10.1111/cns.13805

**Published:** 2022-01-23

**Authors:** Heba Fikry, Lobna A. Saleh, Sara Abdel Gawad

**Affiliations:** ^1^ Department of Histology and Cell Biology Faculty of Medicine Ain Shams University Cairo Egypt; ^2^ Department of Clinical Pharmacology Faculty of Medicine Ain Shams University Cairo Egypt

**Keywords:** cerebellum, curcumin, neuroprotection, Parkinson's disease, rotenone

## Abstract

**Aims:**

Parkinson's disease (PD) is the second most prevalent age‐related neurodegenerative disorder. The cerebellum plays a role in PD pathogenesis. Curcumin has numerous medicinal uses, mostly attributed to its potent antioxidant properties. This study investigated the potential protective influence of curcumin on the cerebellum of albino rats with rotenone‐induced PD.

**Methods:**

Forty adult male albino rats were randomized into four treatment groups: vehicle (group I); rotenone 3 mg/kg/day i.p. injection (group II); rotenone 3 mg/kg/day plus curcumin 30 mg/kg/day i.p. injection (group III); and curcumin 30 mg/kg/day i.p. injection (group IV).

**Results:**

Compared to group I, group II exhibited marked degenerative changes in hematoxylin & eosin‐stained sections and a reduction in Nissl granules in the Purkinje cells of the cerebellum. In group III, the neurotoxic effects in the cerebellum were reduced. Furthermore, the degenerated Purkinje and GFAP‐positive cells increased considerably in group II and were partially reduced in group III versus group II. Compared to group I, rats in group II showed reduced rotarod motor activity, partially restored in group III. Acetylcholine esterase, glutathione, and superoxide dismutase were significantly reduced, and malondialdehyde was significantly increased in group II compared to group I and was partially increased in group III.

**Conclusion:**

Curcumin attenuated neurotoxic effects and degenerative histological changes and alleviated induced oxidative stress in the cerebellar cortex of a PD rat model. Therefore, curcumin dietary supplementation may have neuroprotective effects against the development of cerebellum‐related PD symptoms.

## Graphical abstract

Parkinson's disease is the second most common neurodegenerative condition. Curcumin, a compound with antioxidant properties, ameliorated the damaging effects of Parkinson's disease, which was induced by rotenone administration in the cerebellum of albino rats. Additionally, cholinergic neurotransmission alterations induced by rotenone were suppressed by curcumin.

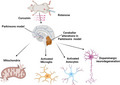

## 1 INTRODUCTION

Parkinson's disease (PD) is the most common neurodegenerative condition after Alzheimer's disease (AD), affecting about 1% of the population over 65 years old. The number of people with PD is expected to rise from 8.7 to 9.3 million by 2030.^1^ PD etiology is unclear, but evidence strongly suggests that chronic neuroinflammation contributes to neurodegeneration.^2^ Moreover, selective loss of dopamine neurons and low striatum dopamine levels occur in PD patients. Dopamine deficiency eventually causes dysregulation of basal ganglia circuits, resulting in clinical motive symptoms, including bradykinesia, restless tremor, rigidity, and postural instability.^3^ Recently, the role of oxidative stress has attracted increasing attention in neurological abnormalities. Therefore, oxidative stress may also play a role in PD pathophysiology.^4^

Pesticide exposure may increase the incidence of PD, as revealed by animal and epidemiological studies. Rotenone is an insecticide and pesticide found in the roots of several plant species.^5^ In humans, nasal, dermal, and inhalation routes are the most significant rotenone exposure risks.^6^ In addition, rotenone is highly lipophilic and crosses biological mucosa, including the blood‐brain barrier (BBB). Thus, the Parkinson's rotenone model has several advantages over other PD models.^7^

The yellow pigment of turmeric and curry (*Curcuma longa* Linn), curcumin, 1,7‐bis (4 Hydroxy‐3 Methoxyphenyl), 1,6‐Heptadiene‐3,5‐Dion, or diferuloylmethane, is a well‐known polyphenolic compound with preventive properties and therapeutic potential for treating neurodegenerative diseases.^8^ Curcumin has a lipophilic property, can pass through cell membranes, and exerts intracellular effects. Curcumin crosses the BBB and is also detected in cerebrospinal fluid. The strong antioxidant properties of curcumin scavenge reactive oxygen species (ROS) and inhibit lipid peroxidation.^9^ In one study, administration of curcumin improved behavioral alterations, oxidative damage, and mitochondrial enzyme dysfunction induced by the administration of rotenone in mice.^10^ Curcumin also restored electrical activity in the hippocampus altered by rotenone.^11^

PD is considered a “classic” basal ganglia disease. Therefore, low dopamine levels in the basal ganglia are the primary focus of experimental and clinical research. However, several studies showed that cerebellar dysfunction and basal ganglia dysfunction are involved in PD‐associated motor and non‐motor symptoms.^12^ Cerebellar dopamine receptors are affected by direct pathogenic alteration via atrophy and denervation. However, greater cerebellar activation occurs during motor execution and during the motor learning process in PD.^13^ Enhanced triggering of the cortico‐cerebellum motor circuitry is hypothesized to compensate for malfunctioning basal ganglia and regulate motor behavior due to hypoactive cortico‐striatal circuitry. Additionally, increased cerebellar activity may result from the defective subthalamic nuclear pathogenic outflow.^12^

The linkage between the basal ganglia and cerebellum provides an anatomical basis for understanding the cerebellum's function in PD and may aid in the development of novel treatment strategies. Thus, the aim of the present study was to determine the potential protective influence of curcumin on the cerebellum in rotenone‐induced PD in albino rats.

## 2 MATERIALS AND METHODS

### 2.1 Chemicals

Rotenone (Catalog number (Cat.) R 8875, Sigma, St. Louis, MO, USA) solution was freshly prepared in dimethylsulfoxide (DMSO) (0.5 mg/ml) and adjusted to pH 7.4 with potassium hydroxide. The solution was immediately used because it is only stable at 25°C for 24 h. Curcumin (Cat.: 458‐37‐7), acetylcholinesterase (AChE) (Cat. C2888), and pyrogallol solution (Cat. 254002) were purchased from Sigma‐Aldrich, Inc. (St Louis, MO, USA). ELISA kits for reduced glutathione (GSH) (Cat.: MBS724319) and lipid peroxidation (malondialdehyde, MDA) (Cat.: MBS738685) were purchased from MyBioSource. Astrocyte‐specialty glial fibrillary acidic protein (GFAP) was purchased from Lab Vision Corp, Medico Co., Egypt (Cat. # MS‐280‐B0), rabbit anti‐tyrosine hydroxylase (TH) polyclonal antibody (Cat #ab117112) was purchased from Abcam, and biotinylated secondary antibody (horse anti‐mouse IgG antibodies) was obtained from Vector Laboratories, Burlingame, CA (BA‐2000‐1.5).

### 2.2 Animals

Forty adult male albino rats weighing 150–200 g (3–6 months) were used. One week before the experiment, the rats were transported to the animal care unit at the Pharmacology Department, Faculty of Medicine, Ain Shams University, for acclimatization to laboratory conditions. The animals were given ad libitum tap water and a commercial diet. The animals were randomly distributed into four experimental groups (10 rats/group). All animal procedures were performed in compliance with the research ethics committee of the Faculty of Medicine, Ain Shams University, Egypt.

### 2.3 Experimental design

In **group I (control)**, rats were treated daily with the vehicle for rotenone and curcumin (DMSO) (1 ml/kg) via intraperitoneal (i.p.) injection. In **group II, the parkinsonism model (rotenone‐treated rats)**, rats were treated with rotenone (3 mg/kg dissolved in DMSO) daily for 60 days.^14^, ^15^ In **group III, the curcumin+rotenone‐treated group**, rats were treated with curcumin (30 mg/kg dissolved in DMSO) and rotenone (3 mg/kg dissolved in DMSO) daily for 60 days.^14^ In **group IV, the curcumin‐only treated group**, rats were treated with curcumin (i.p. 30 mg/kg) and DMSO, 1 ml/kg for 60 days.

Rotenone was adapted based on a previous study showing that chronic exposure to rotenone for 28–60 days impaired locomotion.^15^ This dose of rotenone is not toxic. The LD50 (lethal to half the experimental animals) is between 132 and 1500 mg/kg for rats.^16^ In human clinical trials with doses up to 10 g/day, curcumin was pharmacologically safe.^11^ Animals that died after the i.p. injection were excluded from the study.

### 2.4 Behavioral analysis

The rotarod test, in which animals walk on a rotating drum, is widely used to assess laboratory rodent motor status. The rotarod test measures the duration an animal can stay on the drum in relation to the drum speed. Rats were permitted to adjust their postures to retain equilibrium on the rotary rod for at least 60 s at 12 rpm continuously for three experiments with 5 min intervals. The equipment was washed between testing each animal. The average retention time on the rod was determined.^17^

### 2.5 Tissue collection and preparation

After completing the behavioral evaluation, rats were anesthetized with 2 gm/kg urethane (i.p.) and decapitated. Rat brains were rapidly removed, and the cerebellum was isolated and divided into halves. One half was stored at −80°C for biochemical evaluation, and the other was used for histopathological examination. Moreover, histopathological analyses were used to evaluate neurodegeneration. The sagittal section of the frontal cerebral cortex, striatum, substantia nigra, and hippocampus were also examined in all groups. For histopathological examination, isolated tissues were fixed immediately in 10% neutral‐buffered formalin, processed using a graded ethanol series, and embedded in paraffin. Paraffin sections of 5–7 µm thickness of cerebral and cerebellar hemispheres were stained with hematoxylin and eosin (H&E) stain^18^ for routine examination. The Cresyl fast violet stain (Nissl stain) was used to stain Nissl granules in the Purkinje cells’ perikarya^19^ for paraffin sections of the cerebellar tissue.

### 2.6 Immunohistochemical analysis of TH and GFAP

TH immunostaining within the substantia nigra pars compacta (a marker for dopaminergic neurons) and GFAP immunostaining within the cerebellum (a marker for astrocytes) were performed. Deparaffinized 5‐µm thick paraffin sections placed on positively charged slides were incubated in 10% hydrogen peroxide in absolute methanol (10 min). To unmask performed antigen, the sections were heated in 0.01 mol/l citrate buffer (pH: 6) in a water bath in the microwave for 30 min. The slides were washed in phosphate‐buffered saline (PBS) at pH 7.4 for 5 min before incubation at 4°C for 18–20 h with primary anti‐GFAP antibody (mouse monoclonal antibody, 1/100 diluted in 0.2% Triton X‐100 + 5% goat serum solution), and another slide was treated with primary anti‐phospho‐TH antibodies (rabbit polyclonal antibody, 1/50 diluted in 0.1% Triton X‐100 + 3% horse serum solution). After washing in PBS, the sections were incubated with the appropriate biotinylated secondary antibody (horse anti‐mouse IgG antibodies, 1:500 diluted in PBS containing 0.05% Triton‐X‐100 and 2.5% horse serum) for 1 h at room temperature before being rinsed again in PBS. Streptavidin peroxidase was applied at room temperature for 10 min before rinsing with PBS. To localize and observe the immunoreaction, 3, 3'diaminobenzidine (DAB) hydrogen peroxide was used as a chromogen. Finally, the immunostained sections were counterstained with Mayer's hematoxylin GFAP‐positive cells appeared brown in the cytoplasm of astrocytes. Negative control sections were analyzed by excluding the GFAP antibody.^20^

### 2.7 Morphometric analysis

Samples were analyzed with the Leica Qwin V.3 image analyzer in the Histology and Cell Biology Department, Faculty of Medicine, Ain Shams University. A blinded independent observer examined the slides and took measurements. For each animal, ten different microscopic fields were examined from the substantia nigra pars compacta and cerebellum cortex. Then, the substantia nigra pars compacta boundaries were delineated. From each section, five non‐overlapping fields at 400× were used to calculate the mean number of TH‐positive neurons with visible nuclei,^21^ the number of degenerated Purkinje cells in H&E‐stained cerebellar sections,^22^ and the mean number of positive GFAP immunostained astrocytes/field^23^ for each specimen.

### 2.8 Biochemical measurements in tissue homogenates

For biochemical evaluation, brain tissues from all groups were preserved in PBS at −80°C. The tissues were minced, homogenized, and cooled by the tissue lysate machine with PBS (100 mg tissue/ml). Samples were centrifuged for 15 min at 1500× g (or 5000 rpm), and then the supernatant was collected and stored at −80°C until assayed. Cerebellar homogenates were used for measuring AChE, lipid peroxidation (by measuring MDA), reduced GSH, and superoxide dismutase (SOD).

### 2.9 Effect on acetylcholinesterase activity

AChE activity was measured using an established method modified by Ellman et al.^24^ Absorbance was measured at 412 nm. PBS and acetylthiocholine iodide were utilized as reagents. The reaction was stopped with a solution of 5.5′ dithiobis‐2‐nitrobenzoic acid‐phosphate‐ethanol incubated at 38°C.

### 2.10 Effect on lipid peroxidation by measuring malondialdehyde

Lipid peroxidation forms MDA as a natural byproduct. Thus, a competitive immunoassay technique was used to quantitatively measure MDA in tissue homogenates. The MDA‐horseradish peroxidase (HRP) antibody conjugate was added to induce an enzymatic reaction. The color intensity was measured spectrophotometrically. To calculate MDA concentrations, standard curves were created.

### 2.11 Effect on reduced glutathione

GSH levels in tissue homogenates were determined using a rat GSH ELISA kit. The GSH‐HRP conjugate was added to initiate the enzymatic response. The strength of color, inversely proportional to the reduced amounts of GSH, was calculated spectrophotometrically. Standard curves were generated to calculate GSH concentrations.

### 2.12 Effect on superoxide dismutase

SOD was determined by pyrogallol oxidation inhibition.^25^ An aliquot of the diluted specimen (100 μl) was mixed with 25 μl pyrogallol (24 mmol/L prepared in HCl) to make a volume of 3 ml with Tris HCl (0.1 M, pH 7.8). Absorption changes at 420 nm were measured with a spectrophotometer at 1 min intervals for 3 min. The amount of enzyme that inhibits 50% of pyrogallol autooxidation is a unit of SOD activity. SOD activity was expressed as U/mg.

### 2.13 Statistical analysis

The Statistical Package for the Social Sciences for Windows (v. 26; IBM Corp., Armonk, NY, USA) was used to analyze the data. Data were expressed as the mean and standard error of the mean (SE). Tests of normality (Shapiro–Wilk) were used for data distribution assessments. Parametric tests were performed on normally distributed data. One‐way analysis of variance (ANOVA) was used to compare various groups, followed by Tukey's multiple comparison test. Significance was accepted at *p* < 0.05. GraphPad® Prism Statistical Package Version 9 (2020) was used to visualize the data.

## 3 RESULTS

We included ten rats per group. However, two rats died in the rotenone group (group II) and two rats failed to develop PD symptoms. One rat died and one rat failed to develop PD in group III. No deaths occurred in the other groups. Due to the mortality in groups II and III, additional rats were utilized to keep the number of animals constant (*n* = 10). All rats were evaluated after they finished the treatment procedure.

### 3.1 Effects of curcumin on the rotarod test

**FIGURE 1:**
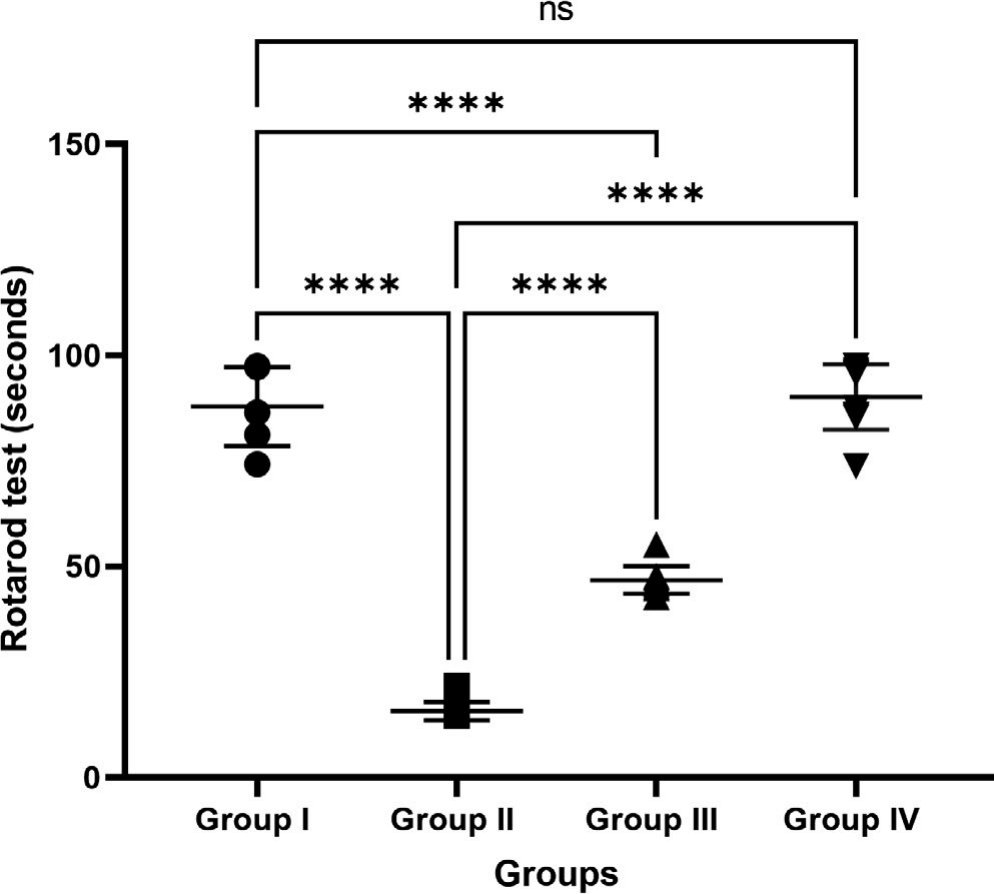
Effect of the tested drugs on rotarod performance in different groups. Values are presented as mean ± standard errors (number of rats = 10). ns = non‐significant, ****p < 0.0001

The effects of curcumin on rotarod performance are shown in Figure 1. One‐way ANOVA revealed significant differences in rotarod performance between groups [F (3, 36) = 311.3, *p* < 0.0001]. A Tukey's post hoc test revealed that the time spent in motor and balance coordination on the rotarod in group II (15.80 ± 0.69s) significantly decreased (*p* < 0.0001) compared with that in group I (87.85 ± 2.95 s) and group IV (90.14 ± 2.46 s). Interestingly, group III (46.75 ± 1.03 s) spent significantly (*p* < 0.0001) more time in motor and balance coordination on the rotarod compared to group II. Rotarod times in groups I and IV were not significantly different (*p* > 0.05).

**FIGURE 2:**
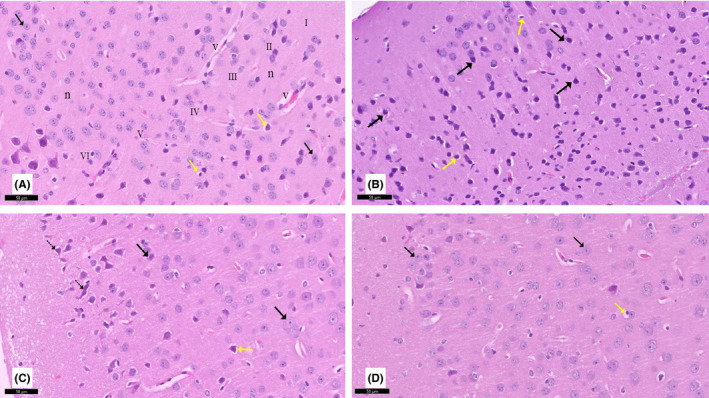
A section of the cerebral cortex stained with H&E viewed at the original magnification (×200). (A) Control rats showed six layers of the cerebral cortex. These layers are: molecular layer (I), outer granular (II), outer pyramidal (III), inner granular (IV), inner pyramidal (V), and multiform layers (VI). Cortical neurons (↑) appear with rounded vesicular nuclei. The neuropil (n) contains astrocytes (yellow ↑) with sharply demarcated nuclei and blood vessels (v) with a narrow perivascular space. (B) Rotenone‐treated rats showed degenerated shrunken neurons (↑) with pyknotic nuclei. Notice hypertrophied astrocytes (yellow ↑). (C) Curcumin‐treated rats (group III) showed most of the cortical neurons (↑) with rounded vesicular nuclei with prominent nucleoli, except for pyknosis in some neurons (dotted arrow) and astrocytes (yellow ↑) with sharply demarcated nuclei. (D) Curcumin‐only treated rats appeared nearly identical to control rats

**FIGURE 3:**
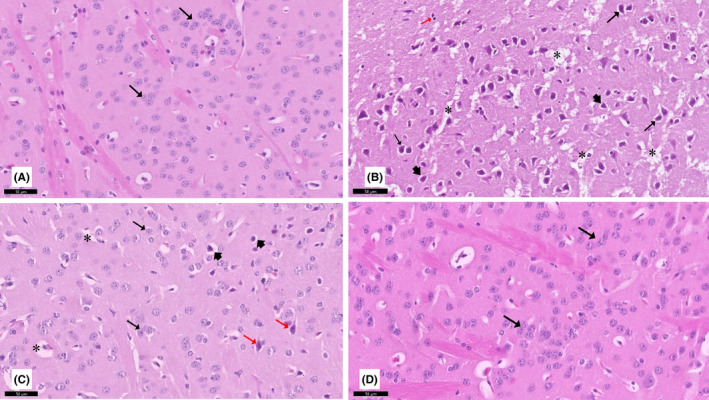
A section of the striatum stained with H&E viewed at the original magnification (×200). (A) Control (group I) showed round to oval neurons (↑) with prominent nucleoli and pale basophilic cytoplasm. Rotenone‐treated rats showed marked neurodegenerative changes. Most of the neurons appeared with an irregular shape and deeply acidophilic cytoplasm (↑) as well as swelling of some neurons (red↑), along with marked neuropil vacuolation (*), and hypertrophied astrocytes (arrowhead) are observed (C) Curcumin‐treated rats showed fewer degenerative changes (red↑) and neuropil vacuolation (*). (D) Curcumin‐only treated rats appeared nearly identical to control rats

### 3.2 Histological results

#### 3.2.1 Hematoxylin & eosin (H&E) stained sections

**FIGURE 4:**
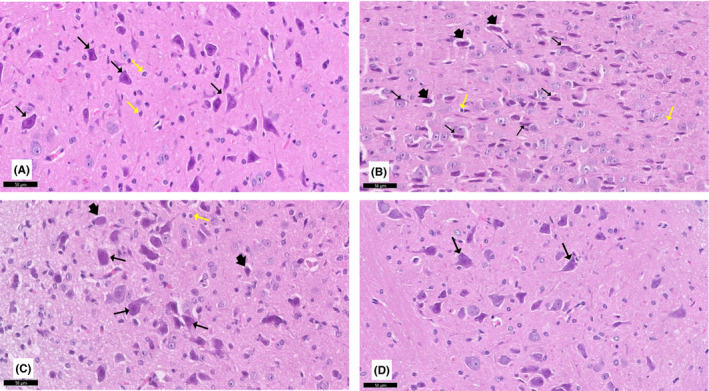
A section of the substania nigra compacta stained with H&E viewed at the original magnification (×200). (A) Control rats show large neurons (↑) with dark basophilic cytoplasm. Astrocytes (yellow ↑) appear with normal organization and strongly defined nuclei. (B) Rotenone‐treated rats’ substantia nigra exhibited strong neuronal loss. Neurons are deeply stained with cytoplasmic inclusions of Lewy bodies (↑) as well as swelling of neurons (arrowhead). Curcumin‐treated rats showed that most neurons (↑) were similar to those of controls and fewer swelled neurons (arrowhead). (D). Curcumin‐only‐treated rats displayed similar features as the vehicle control group

**FIGURE 5:**
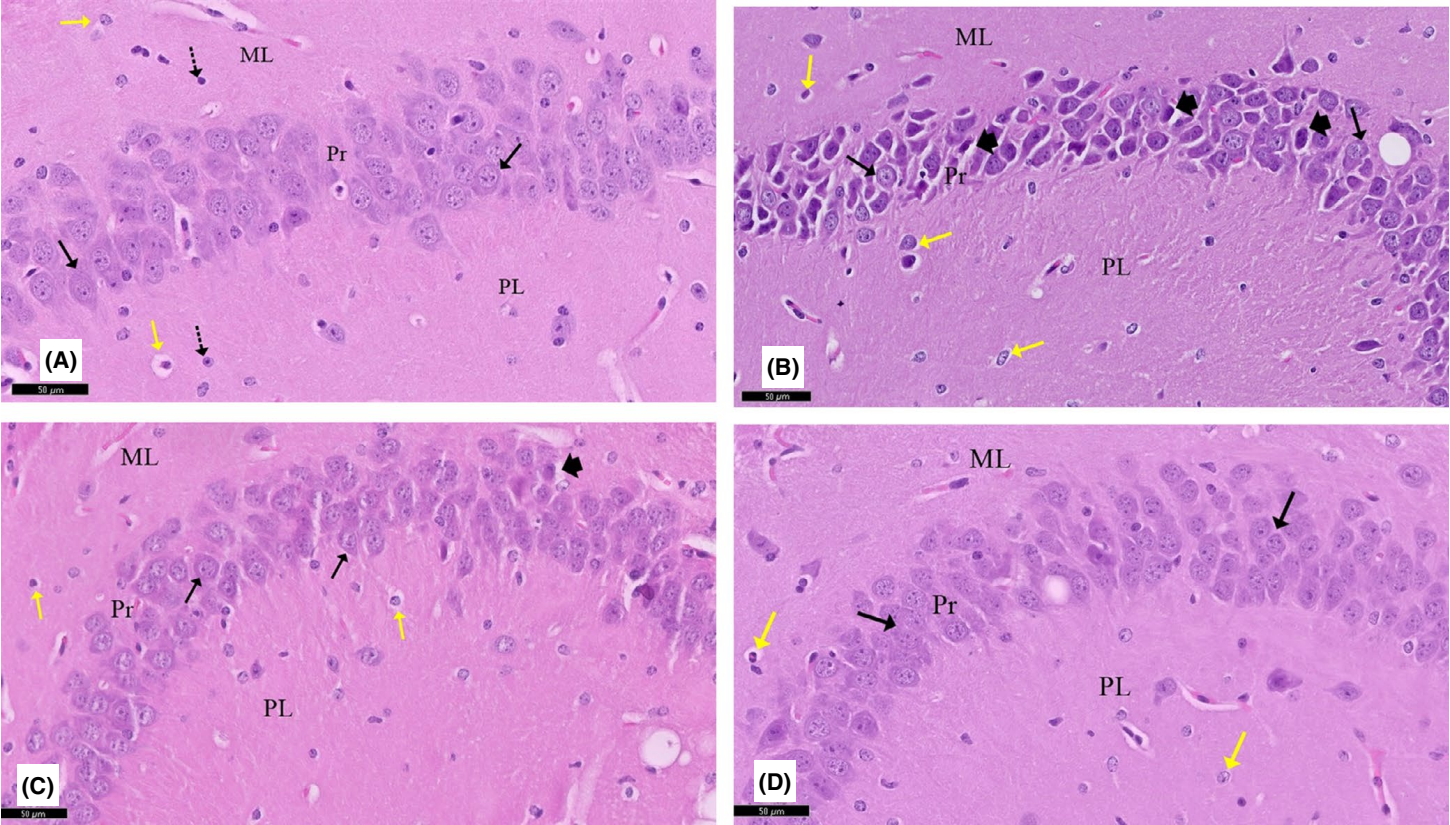
A section of the CA3 area of the hippocampus stained with H&E viewed at the original magnification (×200). (A) Polymorphic (PL), pyramidal (Pr), and molecular (ML) layers are visible in control rats. The Pr layer is made up of three or four rows of densely packed tiny pyramidal cells (↑) with large vesicular nuclei, conspicuous nucleoli, and sparse cytoplasm. There are astrocytes (yellow ↑) and highly stained interneurons (dotted arrow) that could be seen. (B) The CA3 region of rotenone‐treated rats had reduced pyramidal cells with shrunken highly stained pyknotic nuclei and deeply stained basophilic cytoplasm (arrowhead), as well as perineuronal gaps inside the Pr layer. Some pyramidal cells (↑) with pale stained nuclei were observed. Notice hypertrophied astrocytes (yellow ↑) in the ML and PL. (C) Curcumin‐treated rats showed that most neurons (↑) were similar to those of controls and fewer swelled neurons (arrowhead). (D) Curcumin‐only treated rats appear similar to the control group

**FIGURE 6:**
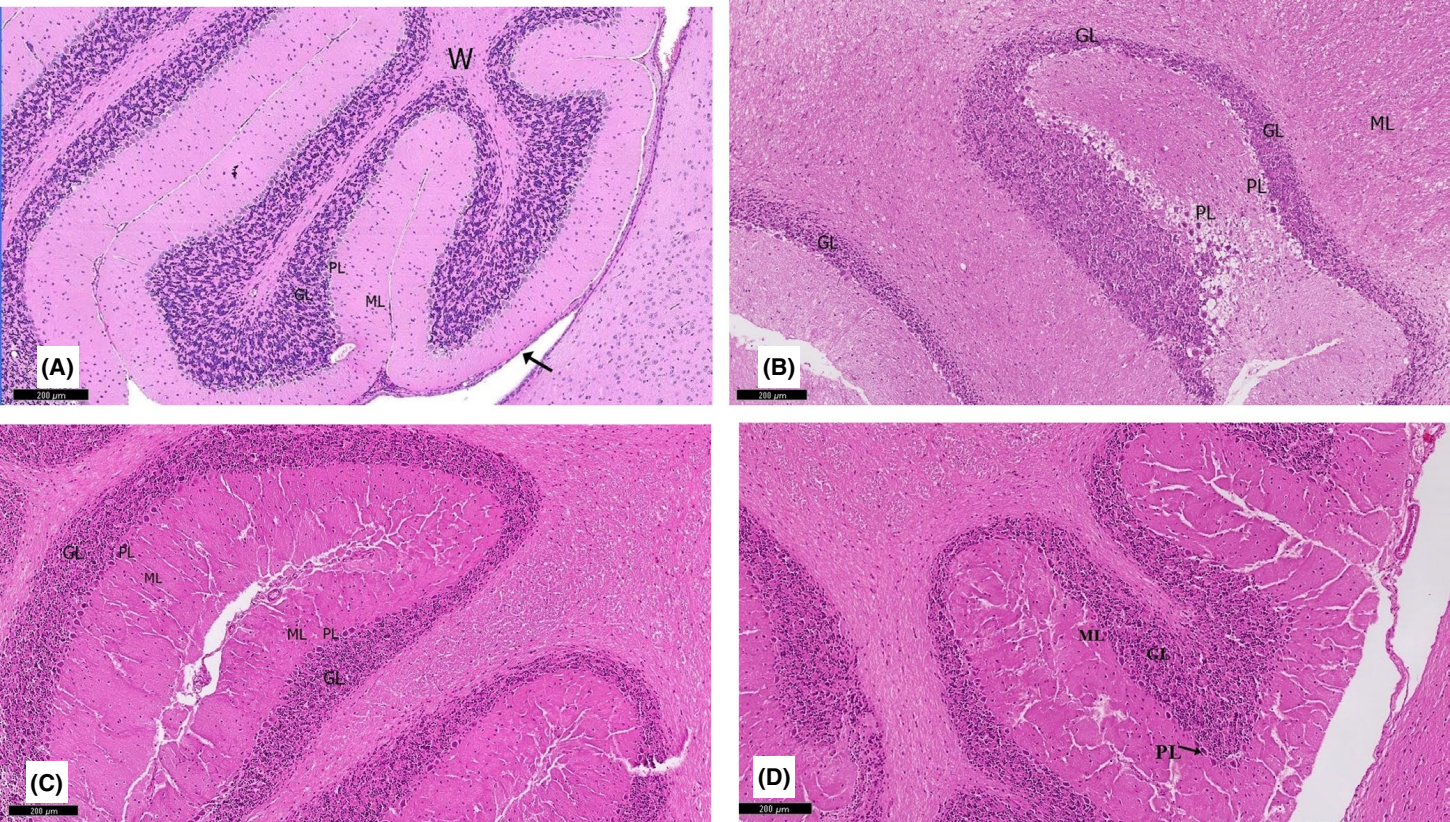
A section of the cerebellum stained with H&E is viewed at the original magnification (×100). (A) Control rats show the normal architecture of folia of the cerebellar cortex, separated by narrow sulci. Each folium consisted of the mantle of the cerebellar cortex with a core of white matter (W). The covering pia mater (↑) can also be observed. (B) Rotenone‐treated rats show a disturbance in the linear organization of the molecular (ML), Purkinje (PL), and the granular layers (GL). (C) Curcumin‐treated rats exhibit a largely normal appearance of the cerebellar cortex. (D) Curcumin‐only treated rats show similar features as the control group

To validate the effectiveness of this rat model of PD produced by rotenone, histological examination was performed primarily on the cerebral cortex, striatum, substantia nigra, and hippocampus, as shown in Figures 2, 4, 5, and 6, respectively.

#### 3.2.2 Cerebral cortex

H&E‐stained sections from control group I revealed that the cerebral cortex consisted of six distinguished layers from the outside inward with no sharp boundaries. These layers were: a molecular layer formed of fibers traveling parallel to the surface with relatively few cells, outer granular, outer pyramidal, inner granular, inner pyramidal, and multiform layers. Cortical neurons had a normal appearance, with rounded vesicular nuclei, pale basophilic cytoplasm, and peripheral processes. The neuropil contained astrocytes with sharply demarcated nuclei, and blood vessels with a narrow perivascular space were seen. Rotenone‐treated rats showed degenerated shrunken neurons with dark cytoplasm and pyknotic nuclei. Notice hypertrophied astrocytes were also seen. Curcumin‐treated rats showed improvement in almost all layers, except some neurons, which appeared with pyknotic nuclei. Curcumin‐only‐treated rats displayed similar features as the control group (Figure 2).

#### 3.2.3 Striatum

The control group's striatum exhibited normal neuronal architecture. Neurons showed round to oval shape with a lesser intensity of staining with clear nuclei. However, in the striatum of the PD rat model, deeply acidophilic irregularly shaped neurons along with vacuolation of neuropils, swelling of neurons, and neuronal degeneration were observed. These histological changes were reduced in the striatum when the rotenone model rats were treated with curcumin. Curcumin‐only‐treated rats displayed similar features as the control group (Figure 3).

#### 3.2.4 Substantia nigra pars compacta

Normal substantia nigra neurons with nerve cells that are generally multipolar, stellate, or pyriform were seen in sections from the control group. These cells have a basophilic cytoplasm. Astrocytes with clearly defined nuclei and typical organization emerged. Rotenone‐treated rats’ substantia nigra displayed high neuronal loss. The cytoplasmic inclusions of Lewy bodies were darkly stained in neurons, and neuronal swelling was observed. When the rotenone model rats were given curcumin, these histological alterations in the substantia nigra were minimized. Curcumin‐only‐treated rats displayed similar features as the control group (Figure 4).

#### 3.2.5 Hippocampus

Sections from the control group showed the normal histological structure of the hippocampus. The CA3 region is divided into three layers: polymorphic, pyramidal, and molecular. The pyramidal layer is made up of three or four rows of tiny pyramidal cells with vesicular nuclei, conspicuous nucleoli, and sparse cytoplasm. Rotenone‐treated rats showed pyramidal cells of the CA3 with signs of degeneration. The pyramidal cells had acidophilic cytoplasm, and many cells were condensed and contracted, with acidophilic cytoplasm and pyknotic nuclei. When the rotenone model rats were given curcumin, these histological alterations in the hippocampus were minimized. Curcumin‐only‐treated rats showed similar features as control rats (Figure 5).

#### 3.2.6 Cerebellum

**FIGURE 7:**
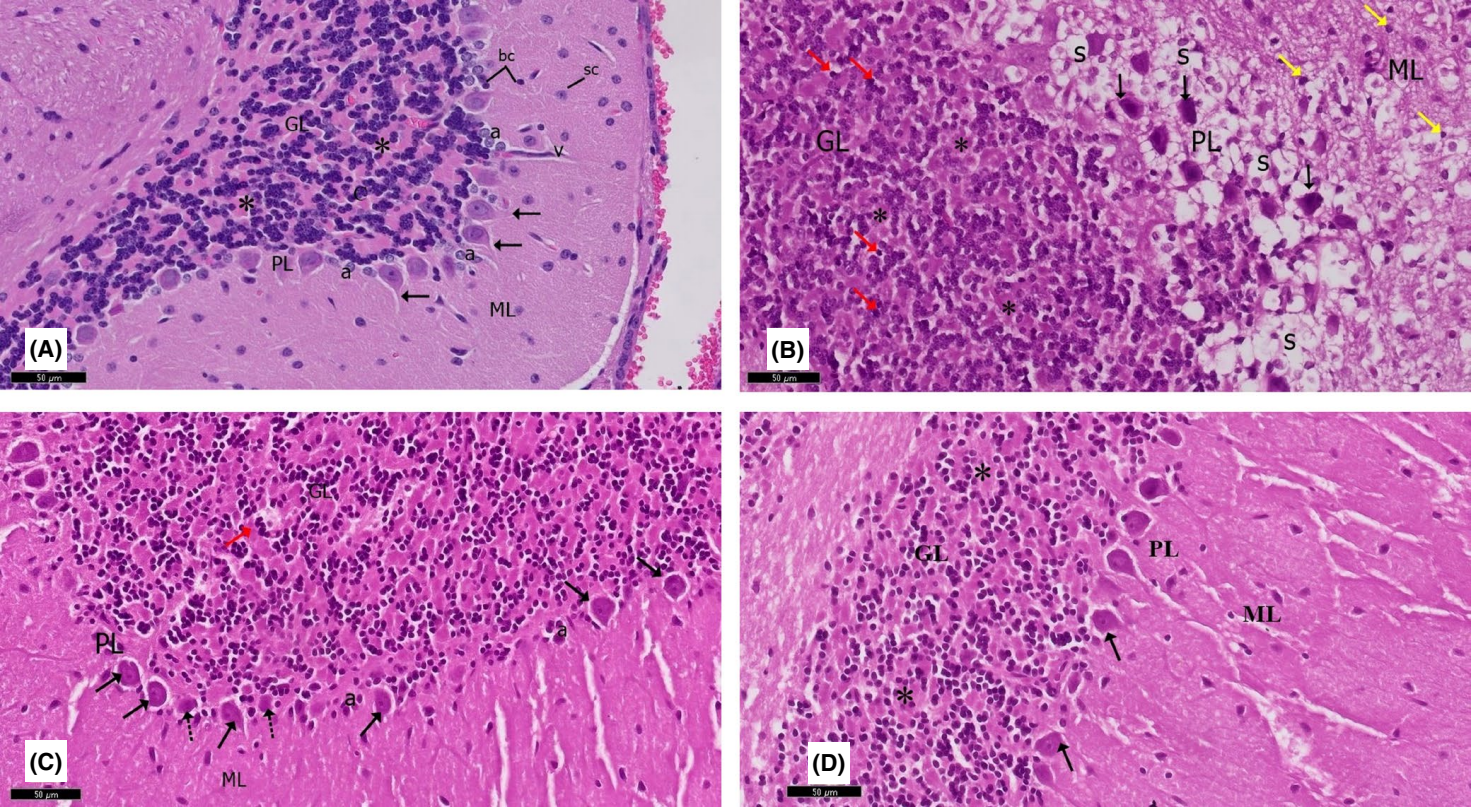
A section of the cerebellum stained with H&E is viewed at the original magnification (×200). (A) Rat cerebellar tissue showing the molecular layer (ML) formed of small stellate (Sc) and basket cells (bc). Purkinje cells of the Purkinje layer (PL) contained one row of flask‐shaped Purkinje cells with apical dendrites (↑). The granular layer (GL) comprised small, tightly packed granule cells. Note the presence of clear spaces of cerebellar islands (*) and Bergmann astrocytes (a). (B) Rotenone‐treated rat showing a disturbance in the linear organization of the middle PL, which showed prominent spongiosis (S) as numerous vacuolated areas. Purkinje cells (↑) are irregular, distorted, and shrunken. Cells of the GL appear shrunken with deeply stained nuclei (red↑). Wide in‐between spaces and loss of cerebellar islands (*) can be observed. The ML shows cells with deeply stained pyknotic nuclei (yellow ↑). (C) Curcumin‐treated rat showing a largely normal appearance. The PL retained a normal linear organization, and most cells (↑) contained rounded vesicular nuclei with prominent nucleoli. The granule cells in the GL and ML have a similar appearance to the controls. Note the presence of Bergmann astrocytes (a) and few Purkinje cells with a fragmented nucleus and eosinophilic cytoplasm (dot arrow). (D) Curcumin‐only‐treated rats exhibit similar features as the control group

**FIGURE 8:**
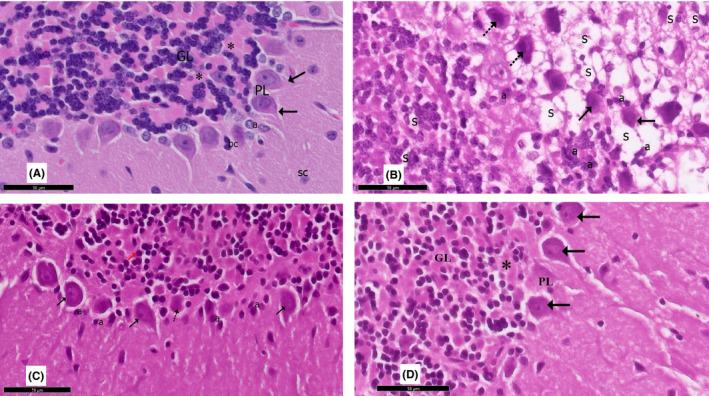
Higher magnification of a previous section stained with H&E viewed at the original magnification (×400)

The effects of curcumin on the cerebellum of rotenone‐induced parkinsonism were evaluated by H&E‐stained sections. Histological findings are presented in Figures 6, 7, and 8. Control group I and curcumin‐treated group IV had normal cerebellar histological structure. The outer molecular layer, the middle Purkinje layer, and the inner granular layer were all part of the cerebellum's grey matter. The outer molecular layer consisted of nerve fibers that were numerous but sparsely distributed, while basket cells were positioned near Purkinje cell bodies and blood vessels deep in the tissue. At the molecular layer's junction with the granular cells, the Purkinje had huge flask‐shaped cells in a single row. Bergmann astrocytes were noted to have pale nuclei and pale cytoplasm and were present in the granular cell layer's superficial region and between the Purkinje cells. A dense population of small granular cells with highly stained nuclei and non‐cellular clear spaces sat between the cells in the granular cell layer, making it appear as if cerebellar islands were present.

Rats treated with rotenone (group II) had marked degenerative alterations in the Purkinje layer. The linear organization of the Purkinje cells was disturbed with marked disarrangement. Purkinje neurons disappeared entirely in many areas leaving empty spaces. The Purkinje cells were deformed, lost their characteristic pyriform shape, appeared shrunken with an irregular shape, and contained deeply stained cytoplasm and barely identified nuclei. Some cells had deeply acidophilic cytoplasm and dark stained shrunken nuclei. The molecular layer showed some cells with deeply stained pyknotic nuclei. Many Bergmann astrocytes and numerous vacuolated areas were observed in all layers.

The cerebellar cortex of curcumin +rotenone‐treated rats (group III) showed marked regression in the cerebellar cortex's neurotoxic effect. H&E‐stained sections revealed a histological pattern similar to the control group. A vast, sparse population of round and elongated neurons was present in the outer molecular layer. The Purkinje cells were grouped into a single row between the molecular and granular layers and retained their normal linear organization and characteristic pyriform shape. They had large rounded vesicular nuclei, prominent nucleoli, and slightly basophilic cytoplasm. The next granular layer contained compactly disposed rounded cells with spherical dark nuclei and scanty pale acidophilic cytoplasm. Cerebellar glomeruli were also observed between the granular cells.

### 3.3 Nissl‐stained section

**FIGURE 9:**
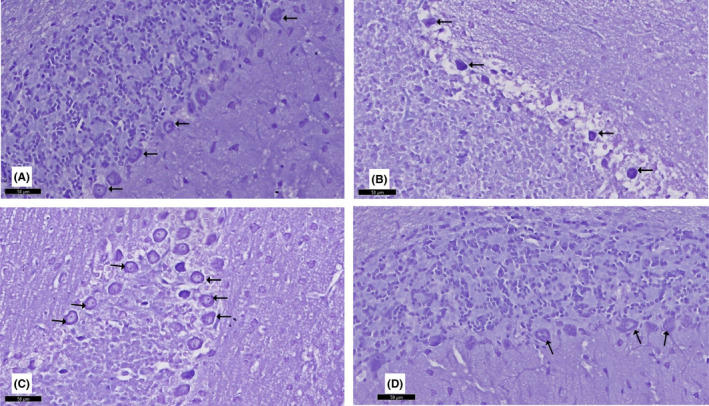
A section in the cerebellum stained with Nissl stain is viewed at the original magnification (×200). (A) Control rats and (D) curcumin‐only‐treated rats show purple Nissl granules (↑) in the perikarya of the Purkinje cells. (B) Purkinje cells appear with an apparent reduction in Nissl granules (↑), giving the cytoplasm a faint appearance in rotenone‐treated rats. (C) Most Purkinje cells show an apparent increase in Nissl granules (↑) in curcumin‐treated rats

The Nissl‐stained sections of the cerebellar cortex are presented in Figure 9. In group I (control group) and group IV (curcumin only), the Purkinje cells exhibited coarse Nissl granules around central vesicular nuclei. Nissl‐stained sections of group II revealed an apparent reduction in Nissl granules, giving the cytoplasm a faint appearance in the Purkinje cells. In group III, more Nissl granules in the Purkinje cells were detected compared to the rotenone‐treated group.

### 3.4 Immunohistochemically stained sections

#### 3.4.1 TH immunohistochemically stained sections

**FIGURE 10:**
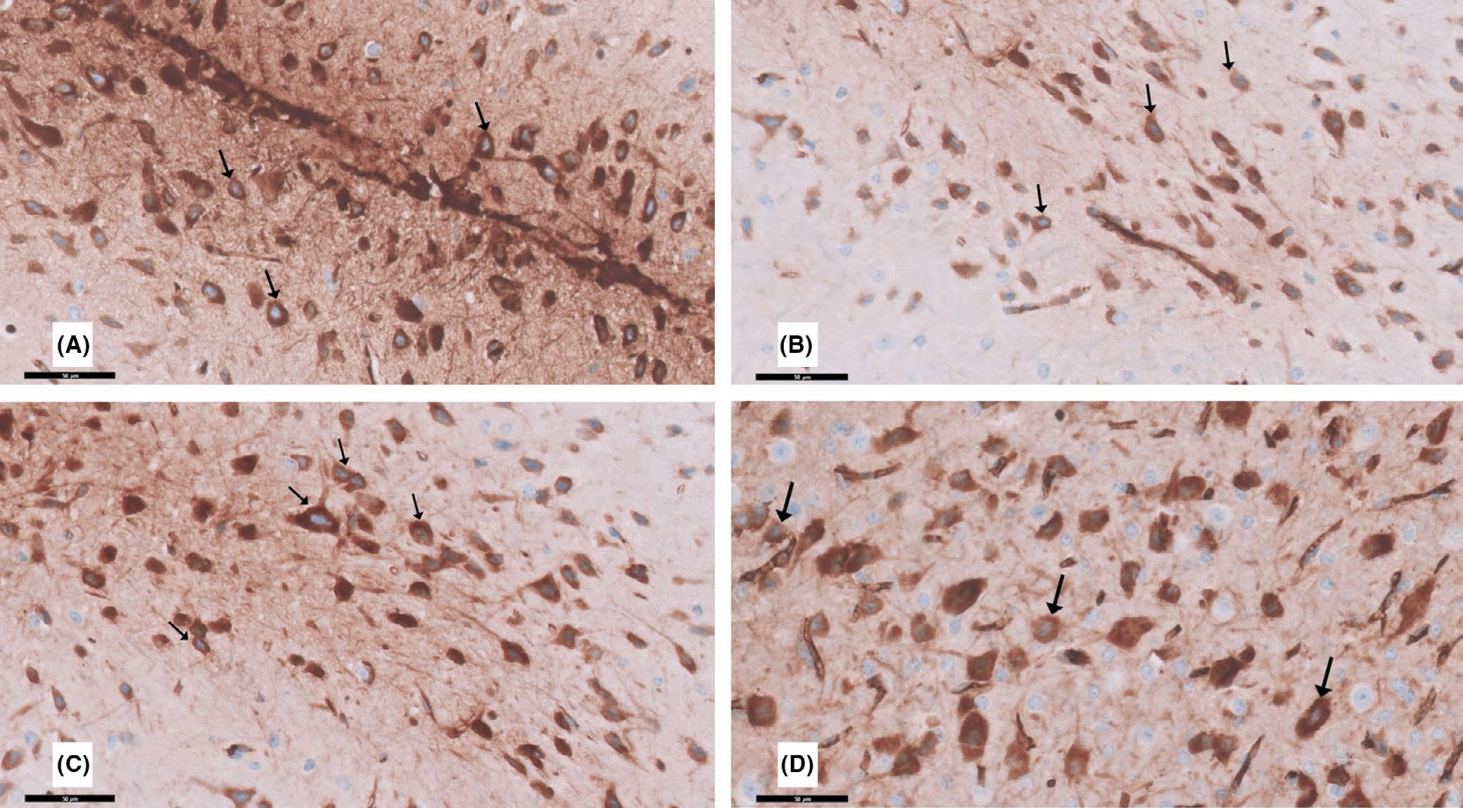
A section in the substantia nigra pars compacts stained with tyrosine hydroxylase (TH) immunohistochemistry is viewed at the original magnification (×200). (A) Control rats and (D) curcumin‐only‐treated rats showing that dark brown cytoplasmic granules of TH immunoreactive (↑) cells are scattered. (B) Apparent marked decrease in TH‐positive immunoreactive nerve cells (↑) of the rotenone‐treated rats compared to the control. (C) Moderate apparent increase of TH‐positive nerve cells (↑) in the curcumin‐treated rats compared to rotenone‐treated group II

Immunohistochemical examination of TH‐positive dopaminergic neurons in the substantia nigra pars compacta is needed to clarify the loss of nigrostriatal dopaminergic neurons in the rotenone rat model of parkinsonism. In the current study, Figure 10 shows TH immunoreactivity in the substantia nigra pars compacta. TH brownish cytoplasmic positive immunoreactive cells were distributed across the substantia nigra pars compacta in group I (control). However, the substantia nigra pars compacta of rotenone‐treated rats (group II) displayed few light‐positive brownish cytoplasmic TH immunoreactive cells distributed throughout. Accordingly, immunohistochemical analysis revealed that curcumin restored TH staining in the substantia nigra. When compared to group II, group III showed an increase in the number of TH‐positive neurons in the substantia nigra. Moreover, group IV (curcumin only) appeared nearly the same as group I.

#### 3.4.2 GFAP immunohistochemically stained sections

**FIGURE 11:**
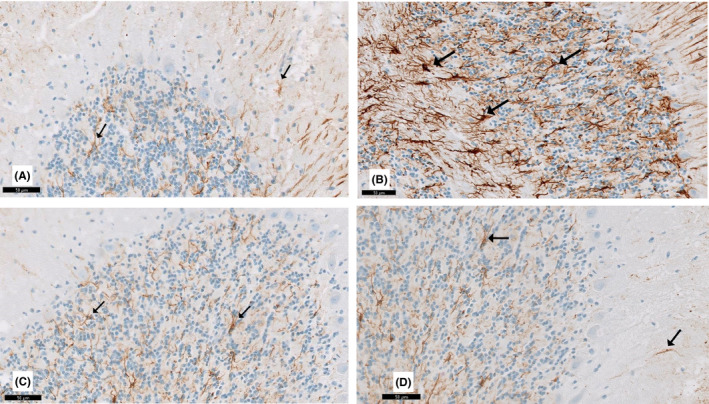
A section in the cerebellum stained with GFAP immunohistochemistry is viewed at the original magnification (×400). (A) Control rats and (D) curcumin‐only‐treated rats showing few GFAP immunoreactive astrocytes (↑) are scattered in the Purkinje cell and granular layers. (B) Apparent increase in GFAP‐positive immunoreactive astrocytes (↑) in all layers of the cerebellar cortex of the rotenone‐treated rats. (C) Few apparent GFAP immunoreactive astrocytes (↑) in the curcumin‐treated rats

GFAP‐positive immunoreactive astrocytes in the cerebellar sections are shown in Figure 11. Groups I and IV showed few GFAP‐positive immunoreactive astrocytes dispersed in the Purkinje and granular layers. In contrast, group II exhibited increased GFAP‐positive cells dispersed in the Purkinje and granular layers. Interestingly, group III exhibited a decrease in GFAP‐positive immunoreactive astrocytes.

### 3.5 Morphometric results

#### 3.5.1 TH‐positive cells in the substantia nigra pars compacta/field

**FIGURE 12:**
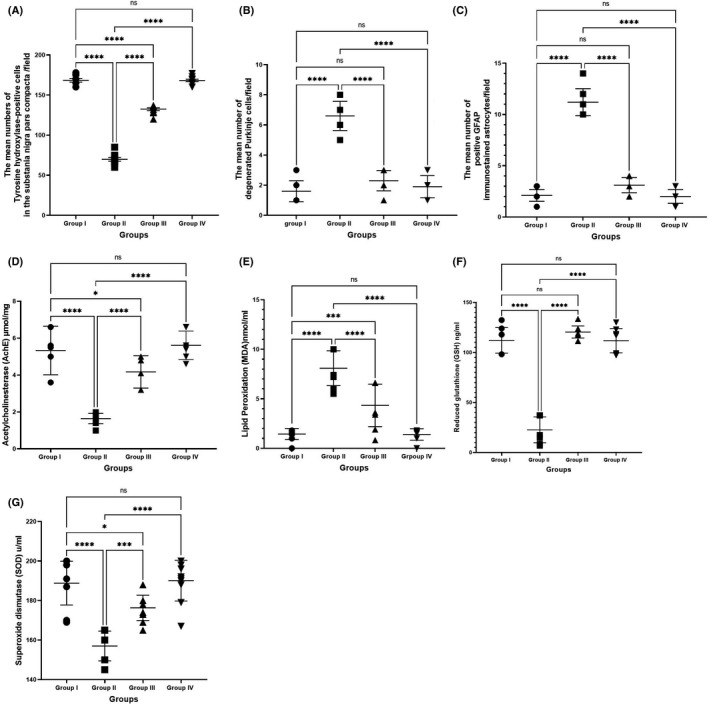
(A) The mean number of TH‐positive cells in the substantia nigra pars compacta/fields in TH immuno‐stained at ×40 magnification in the different groups. (B) The mean number of degenerated Purkinje cells/fields in H&E‐stained cerebellar sections at ×40 magnification in the different groups. (C) The mean number of positive GFAP immunostained astrocytes/fields counted at ×40 magnification in the different groups. Effect of the tested drugs in cerebellar tissue homogenate in the different groups on (D) acetylcholinesterase (AchE) μmol/mg, (E) lipid peroxidation by measuring malondialdehyde (MDA) nmol\ml, (F) reduced glutathione (GSH) ng/ml, and (G) superoxide dismutase (SOD) u/ml. H&E: hematoxylin & eosin. Values are presented as means ± standard errors (number of rats = 10). ns = non‐significant; *p < 0.05, ***p < 0.001, ****p < 0.0001

The numbers of TH‐positive cells in the substantia nigra pars compacta were significantly different [F (3, 36) = 564.7, *p* < 0.0001] between the groups. There was a significant (*p* < 0.0001) decrease in the number of TH‐positive cells in the substantia nigra pars compacta of group II (69.80 ± 2.18) rats compared with the number of cells in groups I (168.4 ± 2.29), III (132.4 ± 1.69), and IV (168.2 ± 1.54). Conversely, the number of TH‐positive cells in the substantia nigra pars compacta in group IV was not significantly different from that in group I (*p* > 0.99) (Figure 12A).

#### 3.5.2 Degenerated Purkinje cells/field

The numbers of degenerated Purkinje cells were significantly different [F (3, 36) = 91.27, *p* < 0.0001] between the groups. There was a significant (*p* < 0.0001) increase in degenerated Purkinje cells in the cerebellar cortex of group II (2.00 ± 0.21) rats compared with the number of cells in groups I (1.60 ± 0.22), III (2.30 ± 0.213), and IV (1.90 ± 0.23). Conversely, the numbers of degenerated Purkinje cells in groups III and IV were not significantly different from group I (*p* = 0.203, *p* = 0.824; respectively). In addition, the numbers of degenerated Purkinje cells were not significantly different in groups III and IV (*p* = 0.66) (Figure 12B).

#### 3.5.3 GFAP‐positive astrocytes/field

A one‐way ANOVA revealed significant differences in GFAP immunostaining between the treatment groups [F (3, 36) = 257.6, *p* < 0.0001]. Positive anti‐GFAP stained astrocytes increased significantly (*p* < 0.0001) in group II (11.20 ± 0.416) compared to group I (2.10 ± 0.17), group III (3.10 ± 0.238), and group IV (2.00 ± 0.21). Similarly, the number of GFAP‐positive astrocytes in group III was significantly higher compared with that in group IV (*p* = 0.037). The number of GFAP‐positive astrocytes in group I was not significantly different from that in groups III and IV (*p* = 0.06, *p* = 0.99; respectively) (Figure 12C).

### 3.6 Biochemical results

#### 3.6.1 Acetylcholinesterase activity

AChE activity was significantly different between the four treatment groups according to one‐way ANOVA [F (3, 36) = 41.16, *p* < 0.0001]. AChE activity in the rotenone‐treated rats (group II) (1.63 ± 0.08) was significantly lower than that in group I (5.33 ± 0.41) and IV (5.61 ± 0.24) (*p* = 0.03, *p* = 0.005; respectively). AChE activity in group III (4.17 ± 0.27) was significantly (*p* < 0.0001) higher than that in group II. AChE activity in groups III and I were not significantly different (*p* = 0.89) (Figure 12D).

### 3.7 Antioxidants and oxidative stress markers in cerebellar tissue

#### 3.7.1 Lipid peroxidation (MDA) levels

One‐way ANOVA demonstrated significant differences in MDA levels between the treatment groups [F (3, 36) = 48.03, *p* < 0.0001]. Tukey's post hoc test revealed that MDA levels were significantly higher in group II (8.09 ± 0.55) compared to group I (1.43 ± 0.17, *p* < 0.0001), III (4.33 ± 0.67, *p* < 0.0001), and IV (1.39 ± 0.18, *p* < 0.001). MDA levels in group I were not significantly different than those in group IV (*p* = 0.99) (Figure 12E).

#### 3.7.2 Glutathione levels

One‐way ANOVA showed significant differences in GSH levels between the four groups [F (3, 36) = 166.6, *p* < 0.0001]. Tukey's post hoc test revealed that GSH levels in group II (22.64 ± 4.08) were significantly lower than those in groups I (112.14 ± 4.03, *p* < 0.0001), III (120.47 ± 1.91, *p* < 0.0001), and IV (120.47 ± 1.91, *p* < 0.0001) as shown in Figure 12F. No significant differences in GSH levels were detected between groups III, I, and IV (*p* = 0.368 and *p* = 0.335, respectively). Also, GSH levels in groups IV and I were not significantly different (*p* = 1.00).

#### 3.7.3 Superoxide dismutase levels

One‐way ANOVA revealed significantly different cerebellum SOD levels between the treatment groups [F (3, 36) = 28.83, *p* < 0.0001]. SOD levels in group II (157.00 ± 2.38) were significantly lower compared to those in groups I (188.80 ± 3.50, *p* < 0.0001), III (176.30 ± 2.02, *p* < 0.001), and IV (190.10 ± 3.26, *p* < 0.0001). Also, SOD levels in group III were significantly lower compared with those in groups I and IV (*p* = 0.05 and *p* = 0.008, respectively). SOD levels in groups I and IV were not significantly different (*p* = 0.98) (Figure 12G).

## 4 DISCUSSION

Cerebellar interactions with the basal ganglia indicate the cerebellum's key role in the development of PD.^14^ According to Wu and Hallett, parkinsonism is possible in primary cerebellar disorders caused by the involvement of the cerebello‐thalamocortical circuit and resulting basal ganglia dysfunction.^12^

Our study revealed neurochemical and histopathological changes in the cerebellum in a rat model of PD induced by rotenone. This may change the disease treatment strategy, which depends mainly on restoring dopamine levels in the basal ganglia. In the current study, we examined the beneficial impact of curcumin in rotenone‐induced PD in the cerebellum of albino rats.

Animal pharmacological models serve as invaluable resources for studying the histopathological mechanisms of PD.^26^ Rotenone is commonly used in rodent PD models as it induces the pathological symptoms and motor defects of PD.^27^ Thus, treatment with rotenone is a feasible way of evaluating the effects of new PD therapeutic agents. The main factor contributing to rotenone's neurotoxic effect is the generation of oxidative stress to produce disease‐like pathology.^14^

Damaged dopamine‐producing cells in the brain are the major cause of PD. The pigmented neurons of the substantia nigra pars compacta of the midbrain degenerate and die selectively. As a result, dopaminergic neuron loss in the substantia nigra pars compacta causes terminal degeneration and dopamine depletion in the striatum, essential for appropriate motor function.^28^, ^29^

In this study, the rotenone group's histological data in the striatum and substantia nigra pars compacta confirmed neuronal necrosis. Additionally, there was a significant decrease in neuronal positivity to TH, indicated by immunohistochemistry in the substantia nigra. Our findings are consistent with previous research.^21^, ^30^, ^31^ In several studies, TH is the standard method for counting dopaminergic neurons.^32^ Shin et al.^29^ found significantly decreased motility, gait impairment, and a 50% drop in TH activity in the substania nigra pars compacta. These alterations support the success of this rat model of PD induced by rotenone.

In the present study, brain damage caused by rotenone was not limited to the nigrostriatal pathway. As evidenced by histological inspection, Rotenone injection also caused major degenerative alterations in the cerebral cortex and hippocampus. Our findings are concordant with those of Abdel‐Salam et al.^33^ who found that rotenone injection caused degenerative alterations in a number of brain locations, including the cerebral cortex, striatum, hippocampus, and substania nigra, resulting from elevated oxidative stress in many brain regions.

Curcumin is a strong antioxidant that minimizes oxidative stress *in vivo* and *in vitro*.^34^, ^35^ Curcumin exerts various beneficial effects in treating and preventing neurodegenerative diseases, such as stroke and AD.^36^, ^37^ Additionally, several studies demonstrated curcumin's ability to inhibit key PD‐associated features, including ROS formation, apoptosis, cytokine production, oxidative damage, and cognitive impairment in cell lines and experimental animals.^38^

In this study, behavioral effects were studied using a rotarod test. Compared to control animals, the mean duration of stay in rotarod‐induced parkinsonism rats was significantly decreased. Group III exhibited a significantly increased duration of stay on the accelerating rotarod compared to group II. Ramkumar et al. observed similar findings; in their study, rotenone administration at low doses (2.5 mg/kg/day) caused dopamine neurodegeneration in the nigrostriatal pathway. In contrast, dose‐based administration of curcumin increased dopamine levels in rotenone‐induced PD rats.^39^ Dopamine is responsible for balance, movement control, and walking.^37^ Bowery et al. reported that Purkinje neurons located in the cerebellum control and organize movement by releasing GABA neurotransmitters.^40^ In addition, Salem et al.^10^ reported that treatment with curcumin prevented the decline in motor activity and muscular strength induced by rotenone and induced restoration of midbrain and striatal dopamine levels. This effect was associated with improved norepinephrine and serotonin levels in the two regions.^41^ Khatri and Juvekar reported that three weeks of curcumin treatment (50, 100, or 200 mg/kg) drastically enhanced behavioral changes and oxidative harm in rotenone‐treated mice.^10^

Most studies suggest a direct relationship between free radicals and microglia, astrocytes, and neurons in neuroinflammatory processes.^42^ ROS play a significant role in apoptosis initiation, which increasing antioxidants can block.^43^ The present study showed that Purkinje cells were affected by rotenone administration. A significant increase in degenerated Purkinje cells was detected in group II compared to groups I and III. This marked increase in damaged Purkinje cells indicates the harmful effect of rotenone on Purkinje neurons. Our results are consistent with other studies.^15^, ^44^ Hasan and colleagues investigated the histological effects of rotenone on the cerebellum of adult Wistar albino rats; rotenone caused cerebellar cortical neural alterations in the Purkinje layer of the cerebellum, resulting in histological damage consistent with motor disability. Furthermore, co‐treatment with curcumin ameliorated the damage to the cerebellum Purkinje layer.^45^ This was explained by Puspita et al.^46^ who stated that ROS might damage major cellular components, such as DNA, membranes, lipids, and carbohydrates. The appearance of deeply acidophilic cytoplasm and dark pyknotic or barely identifiable nuclei might reflect a certain phase of apoptosis or ischemia due to abnormalities in the cerebellar capillary wall cortex, which affects the BBB.^47^, ^48^ In this study, curcumin treatment increased Nissl granules in the Purkinje cells compared to the rotenone‐treated group. Decreased Nissl staining in the rotenone‐treated group may be due to chromatolysis. Hence, the protein‐synthesizing potential of the neurons is lost, leading inevitably to cell death.^49^ Mansouri et al.^44^ showed that the curcumin group had substantially greater Nissl neurons on the left side of the substantia nigra. They concluded that curcumin might prevent the progression of neurotoxic matter.

Immunohistochemical staining confirmed the histological observations in this study. The enhancement of Bergmann astrocytes, indicated by increased expression of GFAP, was apparent after rotenone treatment. Astrocytes react immediately to any nervous system insult by producing neurotoxic substances and GFAP. Thus, GFAP is considered a marker for astrogliosis.^50^ GFAP‐positive cells increased in group II compared to groups I and III. This might be due to a compensatory mechanism following neurodegeneration. A similar process was reported following neurotoxicity. Astrocytes are involved in neuronal homeostasis, synaptic plasticity, and glial cell activation, resulting in a secondary mechanism of cell death or neuroprotective response.^51^ Paul and Borah^48^ reported increased GFAP‐positive cells, leading to positive and negative outcomes. GFAP‐positive cells may protect neural parenchyma against ischemia, inflammation, and neurodegeneration. Unfortunately, glial cells secrete inflammatory cytokines and free radicals, which cause neuronal damage. In this study, curcumin‐treated rats showed a significant decrease in the mean number of anti‐GFAP stained astrocytes compared to group II. Our data generally agree with other studies.^52^, ^53^

Cholinergic input to the cerebellum is critical for modulating cerebellar activities; hence, cholinergic disorders may result in physiological impairment.^54^ Transmitted signals end in cholinergic neurotransmission by cleavage of acetylcholine, generating acetate and choline. This breakage is catalyzed by AChE, part of the α/β‐fold family of proteins. Each AChE molecule may hydrolyze 5000 ACh molecules every second, making AChE one of the most kinetically effective enzymes.^55^

Increasing data indicate decreased AChE activity in numerous brain disorders, including PD and AD.^48^ AChE is also a sensitive enzyme inhibited by increased radical substance formation. Activation of AChE is a critical therapeutic target in treating PD.^14^ Additionally, the primary motor symptoms of PD are mostly attributable to a loss of dopaminergic tone and a resulting imbalance in the regulation of striatal output by dopaminergic and cholinergic neurons. Thus, anticholinergic medicines may help alleviate disease symptoms, especially the related tremor.^33^ Reducing AChE activity is likely to aggravate PD motor characteristics.

Our results indicate that curcumin treatment in a rat model of PD restored cerebellar AChE activity to nearly the same level as controls, providing an argument for its pathogenic influence on brain functions. Our result is consistent with earlier reports.^33^, ^56^ Khadrawy et al.^56^ reported that AChE is sensitive to free radicals that inhibit enzyme activity. Thus, this could explain the present decrease in AChE activity. They reported a decrease in AChE activity, which exaggerates the state of depolarization and excitation by elevating cerebellar ACh through interaction with nicotinic receptors. In the cerebellum, nicotinic acetylcholine receptors mediate the release of glutamate. Another study suggested that reduced AChE activity may result from the enzyme becoming exhausted during the breakdown of elevated ACh. Additionally, the decrease in AChE activity may result from the enzyme's sensitivity to ROS. Salem et al.^41^ reported that curcumin treatment induced significant increases in AChE activity. Our results suggest that curcumin‐induced restoration of cerebellar AChE activity could alleviate a number of the deficits caused by rotenone.

In this study, treatment with rotenone significantly increased MDA and significantly decreased GSH and SOD in group II versus group III. These findings agree with the results of previous studies.^15^, ^16^, ^57^ Hasan et al.^14^ reported that treatment with rotenone increased lipid peroxidation and decreased nitric oxide, GSH, SOD, and catalases, indicating increased oxidative stress in mice. Manjunath et al. reported that rotenone induced significant oxidative stress, indicated by high hydroperoxides, lipid peroxidation, and nitrites *in vivo*.^58^ ROS and mitochondrial oxidative damage are minimized by curcumin. In the mitochondria, curcumin increases the defense efficiency compared to curcumin outside the mitochondria.^14^ Salem et al.^41^ reported that curcumin protection and treatment ameliorated most of the oxidative stress parameters induced by rotenone and improved the histopathological alterations in the two brain areas. Moreover, Khuwaja et al. demonstrated that curcumin helps stop parkinsonism and has therapeutic potential. Khuwaja et al.^59^ used 6‐hydroxydopamine to induce parkinsonism in rats in the right striatum. Three weeks of lesioning decreased behavioral activity in the injured group compared to the sham group, and pretreatment with curcumin provided significant protection. MDA was reduced and GSH, SOD, and catalase levels were increased in animals treated with 80 mg/kg curcumin for 21 days.

Interestingly, in the current study, curcumin alone did not negatively affect the test parameters and histologic structures in the cerebellar tissue, in agreement with other studies.^60^ Based on the results of this study, rotenone causes Purkinje cell death and astrogliosis by increasing oxidative stress in the cerebellar cortex. Administration of curcumin prevented these effects. Furthermore, cholinergic neurotransmission alterations induced by rotenone were suppressed by curcumin, thus confirming the behavioral and histological findings.

## 5 CONCLUSION

This shows that curcumin attenuated the neurotoxic effects and degenerative histological changes in the cerebellar cortex and alleviated oxidative stress in a PD rat model. Thus, curcumin could have a role in therapeutic strategies for cerebellar affection related to PD.

## CONFLICT OF INTEREST

The authors declare that they have no relevant or material financial interests related to the research described in this paper.

## AUTHORS’ CONTRIBUTIONS

The authors confirm contribution to the paper as follows: study conception and design: Dr. Heba Fikry, Dr. Sara Abdel Gawad, Dr. Lobna A. Saleh; data collection: Dr. Heba Fikry, Dr. Sara Abdel Gawad, Dr. Lobna A. Saleh; analysis and interpretation of results: Dr. Heba Fikry; manuscript draft preparation: Dr. Heba Fikry. All authors reviewed the results and approved the final version of the manuscript.

## Supporting information

Supplementary Material

## Data Availability

The data that supports the findings of this study are available in the Supplementary Material of this article.
